# Ultrastructural Aspects of Oocyte Maturation in Dogs, with Comparative Insights from Cats: Current Evidence and Research Perspectives

**DOI:** 10.3390/ani16050798

**Published:** 2026-03-04

**Authors:** Lalith sai Jammula, Malgorzata Ochota, Michal J. Kulus

**Affiliations:** 1 Department of Biotechnology and Food Sciences, University of Teramo, 64100 Teramo, Italy; 2 Department of Reproduction and Clinic of Farm Animals, Wroclaw University of Environmental and Life Sciences, 50-366 Wrocław, Poland; malgorzata.ochota@upwr.edu.pl; 3 Division of Ultrastructural Research, Faculty of Medicine, Wroclaw Medical University, 50-368 Wrocław, Poland; michal.kulus@umw.edu.pl

**Keywords:** oocyte maturation, cytoskeleton, assisted reproductive technologies, germinal vesicle breakdown, in vitro maturation

## Abstract

Reproductive success in dogs and cats remains limited despite advances in assisted reproductive technologies, highlighting the need for a clearer understanding of the ultrastructural processes governing oocyte maturation. This review critically examines current knowledge on the ultrastructural and cytoplasmic mechanisms underlying oocyte maturation in dogs, with particular emphasis on cytoskeletal dynamics, organelle redistribution, mitochondrial activity, and lipid metabolism. Comparative insights from other mammalian species, especially the cat, are integrated to highlight fundamental differences in the timing and regulation of oocyte competence. The evidence suggests that inadequate cytoplasmic maturation, not meiotic progression, is the principal bottleneck affecting fertilization and early embryonic development in dogs. We conclude that future improvements in canine fertility and reproductive technologies will require a shift from predominantly hormonal and nuclear-focused approaches toward strategies targeting oocyte cytoplasmic quality and ultrastructural maturation.

## Simple Summary

Reproductive inefficiency in dogs remains a major challenge in both clinical practice and assisted reproductive technologies. Unlike many other mammals, canine oocytes are ovulated at an immature stage and must complete their maturation after ovulation, a process that is still poorly understood. This review focuses on the ultrastructural and cytoplasmic events that regulate oocyte maturation in dogs, with particular emphasis on cytoskeletal organization, organelle redistribution, and metabolic readiness. By comparing canine oocyte maturation with that of other domestic species, especially cats, we highlight species-specific mechanisms that may explain the limited success of current reproductive technologies in dogs. Understanding these processes is essential for improving fertility management and developing more effective interventions.

## 1. Introduction

Pedigree dogs and cats are increasingly presented for advanced veterinary reproductive care, reflecting intensified selective breeding practices and rising expectations for reproductive success in animals of high genetic, behavioral, or phenotypic value [1]. At the same time, progress in reproductive biotechnologies has expanded the range of clinical interventions available in small animal reproduction, raising expectations for outcomes comparable to those achieved in specialized assisted reproduction laboratories [2]. Despite these advances, reproductive efficiency—particularly in dogs—remains inconsistent, indicating that technological developments have outpaced a mechanistic understanding of species-specific reproductive biology [3].

A major challenge arises from the profound physiological differences between the bitch and the queen, which directly influence reproductive management strategies. The bitch is monoestrous, with long inter-estrous intervals and a prolonged luteal phase that occurs independently of pregnancy [4,5]. Proestrus typically lasts approximately nine days, followed by a similar estrus period and spontaneous ovulation [4]. While this predictability facilitates ovulation timing and pregnancy diagnosis, it also predisposes bitches to pseudopregnancy and complicates interpretation of luteal hormone profiles [5].

In contrast, the queen is seasonally polyestrous and typically an induced ovulator, rapidly re-entering estrus in the absence of copulation-induced ovulation [4,6]. Without ovulation, queens may cycle repeatedly during the breeding season, requiring diagnostic and therapeutic approaches that differ substantially from those used in dogs [1]. Although these reproductive patterns are well documented, their implications for oocyte competence and assisted reproductive outcomes remain insufficiently integrated into mechanistic models of oocyte maturation.

While previous reviews have summarized hormonal regulation and clinical management in carnivore reproduction, fewer have critically examined how species-specific follicular physiology and cytoplasmic maturation events interact to determine oocyte developmental competence. The present review therefore focuses on ultrastructural and cytoplasmic determinants of oocyte maturation in dogs and cats, with the aim of identifying biological constraints that may underlie the variable success of assisted reproductive technologies in these species. In this way, we seek to move beyond descriptive accounts of reproductive physiology and provide a mechanistic framework linking follicular dynamics, organelle organization, and ART outcomes.

Reproductive success in both species ultimately depends on the developmental competence of the oocyte, which determines the fertilization outcome and early embryonic survival [7]. Oocyte competence is not a fixed property but results from coordinated nuclear and cytoplasmic maturation during follicular development and the peri-ovulatory period [3]. Nevertheless, many clinical and experimental approaches continue to emphasize ovulation timing and meiotic progression, often assuming that nuclear maturation adequately reflects overall oocyte quality.

Species-specific differences in oocyte maturation challenge this assumption. In dogs, oocytes are ovulated at the germinal vesicle stage and require prolonged post-ovulatory maturation within the oviduct before reaching metaphase II competence [8]. This characteristic complicates the timing of mating, artificial insemination, and in vitro maturation protocols and is widely regarded as a major factor contributing to the limited success of canine assisted reproductive technologies [2,3,9]. However, the extent to which delayed nuclear maturation alone accounts for reduced developmental competence remains debated, as some studies suggest that cytoplasmic remodeling may continue even after meiotic progression, creating potential discordance between nuclear stage and functional quality.

A critical comparison of published canine in vitro maturation studies reveals substantial variability in outcomes that cannot be explained by a single technical factor. Reported rates of meiotic resumption often approach 50–60% [10,11,12], yet full maturation to metaphase II rarely exceeds 15–30%, even under optimized conditions [2]. Differences in basal media (e.g., TCM-199 vs. SOF) [12,13,14], protein supplementation (Serum vs. BSA) [14,15,16], hormonal additions (FSH, LH, hCG, eCG, Progesterone) [11,12,13,15], and culture systems (drop culture, follicle culture, oviductal co-culture, or isolated oviduct models) [10,14,16,17] have all been associated with modest or inconsistent improvements [2]. Extending culture duration may increase GVBD rates but frequently results in higher degeneration [10,13,16]. These inconsistencies suggest that current protocols fail to replicate the unique endocrine and oviduct-dependent conditions under which canine oocytes complete maturation in vivo. Limited understanding of the functional status of the ovulated GV-stage oocyte, the role of corona radiata cells in maintaining or releasing meiotic arrest, and the contribution of oviductal secretions during the prolonged post-ovulatory interval likely underlie this variability [8,17,18,19,20]. Moreover, donor-related factors, including age, breed, and stage of estrous cycle, have been shown to significantly influence maturation outcomes further complicating direct comparison between studies [13,15,16,21].

In contrast, feline oocytes are ovulated at metaphase II following copulation-induced LH release, although variability in ovarian response and follicular synchrony may still limit reproductive predictability [1,4]. While this earlier acquisition of nuclear maturity facilitates the application of conventional invitro maturation and fertilization protocols, feline folliculogenesis follows a more classical mammalian pattern with greater follicle-oocyte synchrony. This relative predictability has supported the development of effective ART strategies and has positioned the domestic cat as a valuable translational model for wild fields. Nevertheless, even in cats, cytoplasmic factors such as mitochondrial distribution, lipid organization, and metabolic readiness remain important determinants of developmental competence [2].

These interspecies differences therefore highlight not only divergent reproductive timing but also uncertainty regarding which maturational parameters most accurately predict developmental competence.

A central and ongoing debate in reproductive biology concerns the relative contribution of nuclear and cytoplasmic maturation to oocyte developmental competence. While nuclear maturation ensures correct chromosomal segregation, cytoplasmic maturation governs the redistribution and functional readiness of organelles required for early embryogenesis [7]. During meiotic divisions, polar bodies are extruded and must not retain essential cytoplasmic components, as their loss would compromise subsequent development. Accordingly, the presence of a metaphase II spindle alone cannot be considered a sufficient indicator of oocyte quality, particularly in species such as the dog, in which meiotic progression may occur in the absence of complete cytoplasmic maturation [3].

Cytoplasmic maturation involves coordinated spatial and biochemical reorganization of mitochondria, endoplasmic reticulum, cortical granules, and cytoskeletal elements. The cytoskeleton plays a central role in anchoring organelles within the ooplasm and preventing their extrusion into polar bodies, thereby preserving developmental competence [7]. Although disruption of these processes has been associated with impaired oocyte quality in several mammalian models, the extent to which similar mechanisms operate in dog and cats remain insufficiently characterized, limiting direct translation into optimized species-specific ART protocols [3].

Despite advances in ultrastructural and imaging techniques, including transmission electron microscopy, immunofluorescence, and live-cell analyses, the molecular regulation of organelle positioning and cytoskeletal remodeling during oocyte maturation remains incompletely understood in dogs and cats [2,3]. This limitation is particularly relevant given that many assisted reproductive protocols continue to rely on nuclear maturation as a surrogate marker of oocyte competence. Thus, a critical evaluation of cytoplasmic and ultrastructural maturation is necessary to refine current diagnostic criteria for oocyte quality in carnivores.

These knowledge gaps are particularly consequential in carnivores, in which the temporal coordination between nuclear maturation, cytoplasmic maturation, and fertilization is highly species-specific. In dogs, meiotic resumption is delayed until the oocyte reaches the mid-oviduct, and fertilization becomes possible only two to three days after ovulation [8]. In contrast, in cats, meiosis resumes within the ovary following copulation-induced LH release, allowing fertilization to occur shortly after oviductal entry [1,4]. These divergent timelines underscore the importance of cytoplasmic preparedness at the time of fertilization and illustrate why extrapolation from other mammalian models may be misleading in carnivores [3].

This review examines the role of oocyte ultrastructure and cytoplasmic maturation in determining reproductive success in dogs and cats. By integrating ultrastructural, cellular, and physiological evidence and addressing mechanistic limitations underlying current assisted reproductive approaches, this review aims to support improved assessment of oocyte quality, refinement of breeding management, and development of more effective species-specific reproductive technologies in small animal practice. In particular, we emphasize areas where empirical evidence remains limited and propose directions for targeted experimental investigation in carnivore models.

## 2. Ovary and Follicular Development

**Figure 1:**
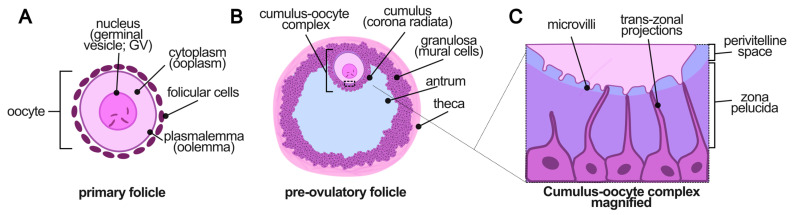
Summary of the basic terminology for mammalian oocytes and related structures. Terms presented below will be used thorough all manuscript. (**A**) **Primary follicle.** The oocyte is enclosed by a single layer of pre-granulosa (follicular) cells, which differentiate into granulosa cells as follicles mature. The oocyte nucleus (*germinal vesicle*, GV) is characteristic of meiotic arrest. In dogs, oocytes are ovulated at an immature **GV stage** and retain an intact nuclear membrane for 48–72 h post-ovulation before undergoing germinal vesicle breakdown (GVBD). In contrast, feline oocytes typically resume meiosis **before ovulation**, progressing to MI–MII stages following the LH surge. (**B**) **Pre-ovulatory follicle.** As follicles enlarge, granulosa cells organize into two functional compartments [22] the *cumulus*, including the corona radiata directly contacting the oocyte, and ref. [9] the *mural granulosa cells (MGCs)* lining the follicular wall. Stromal cells differentiate into *theca interna* and *theca externa*. The antral cavity contains follicular fluid. Species differences include: in dogs, pre-ovulatory follicles reach relatively large diameters and the cumulus–oocyte complex (COC) remains compact with minimal mucification; in cats, COC expansion is moderate but more evident after LH stimulation. (**C**) **Oocyte–cumulus interface.** Between the oocyte and cumulus cells lie two distinct regions: the *zona pellucida (ZP)* and the narrow *perivitelline space (PVS)*. The canine ZP is notably thick and remains stable during prolonged post-ovulatory maturation, whereas in cats its thickness resembles that of other induced-ovulating mammals [3,6,23]. Cumulus cells maintain direct communication with the oocyte via *trans-zonal projections (TZPs)*, which form gap junctions primarily composed of connexin-37 (CX37). These junctions support metabolic and molecular exchange essential for oocyte competence until TZP withdrawal at late maturation [24,25,26]. The oolemma displays microvilli that participate in oocyte–cumulus signaling and later facilitate gamete membrane interactions.

To ensure consistent terminology throughout the manuscript, Figure 1 summarizes the basic terminology for mammalian oocytes and related structures, which will be used throughout the current article.

**Figure 2:**
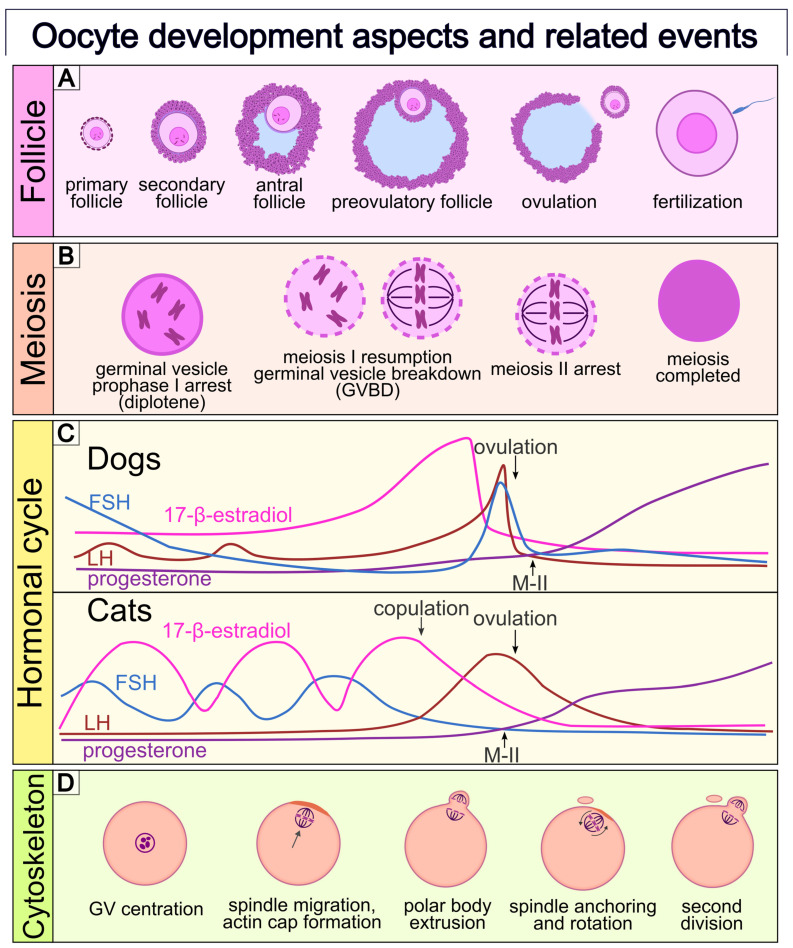
Different aspects of oocyte development. Events and structures depicted at similar horizontal positions occur approximately at the same time. (**A**) **Follicular development and morphology.** Primordial follicles consist of an oocyte surrounded by a single layer of squamous pre-granulosa cells. Upon activation, they develop into primary follicles, characterized by cuboidal granulosa cells. Further growth leads to secondary follicles with multiple layers of granulosa cells. As granulosa cells proliferate and secrete follicular fluid, fluid-filled spaces form between granulosa cell layers and merge to create the antrum, forming antral follicles. Dominant follicles reach the preovulatory stage with a large antrum and a defined cumulus–oocyte complex (COC), which is released into the oviduct after the LH surge. (**B**) **Oocyte nuclear maturation and meiotic progression.** Prior to the LH surge, the oocyte is arrested in prophase I (dictyate stage), with an enlarged nucleus termed the germinal vesicle (GV). Meiotic resumption begins with germinal vesicle breakdown (GVBD), followed by progression through metaphase I (MI), and arrest at metaphase II (MII). In dogs, oocytes are ovulated at an immature GV stage and retain an intact nuclear envelope for approximately 48–72 h post-ovulation before undergoing GVBD. In contrast, feline oocytes typically resume meiosis before ovulation and progress to MI–MII stages following the LH surge. Completion of Meiosis II occurs only after fertilization. (**C**) **Hormonal profiles in dogs and cats.** Schematic representation of peri-ovulatory changes in FSH, LH, 17-β-estradiol, and progesterone. In dogs, a preovulatory rise in progesterone precedes ovulation, which occurs while oocytes are at the MI stage. In cats, ovulation is induced by copulation, triggering an LH surge and ovulation of MII-stage oocytes. Hormone profiles are illustrative and not to scale. (**D**) **Cytoskeletal dynamics.** During the GV stage, cytoskeletal components are distributed throughout the ooplasm. Meiotic resumption involves actin- and microtubule-dependent spindle formation and migration toward the oolemma, accompanied by actin cap formation. The cytoskeleton is essential for asymmetric cytokinesis, extrusion of the first and second polar bodies, and correct positioning and rotation of the meiotic spindle. Figure based on [31].

Folliculogenesis in mammals follows a broadly conserved sequence from primordial follicle activation through primary, secondary, and antral stages, culminating in ovulation (Figure 2). However, in carnivores, and particularly in dogs and cats, several species-specific features distinguish ovarian and follicular development from that described in commonly studied laboratory and livestock species. These differences have important implications for oocyte developmental competence and may contribute to the limited efficiency of assisted reproductive technologies in these species.

In the bitch, follicular development is characterized by relatively large preovulatory follicles and early luteinization of granulosa cells, which begins before ovulation and continues rapidly thereafter [4,5]. This early luteinization alters the endocrine and paracrine environment surrounding the oocyte during the peri-ovulatory period and may influence oocyte–somatic cell communication, as suggested by integrative analyses of canine follicular physiology [2,3]. Although ovulation timing in dogs can be predicted with reasonable accuracy, the functional consequences of early luteinization for oocyte cytoplasmic maturation remain poorly understood. This gap is particularly relevant given that canine oocytes are ovulated at an immature germinal vesicle stage and must complete maturation post-ovulation [8,22].

In contrast, the queen exhibits seasonally polyestrous ovarian activity and ovulates in response to copulation-induced luteinizing hormone release [4,6]. Follicular development in cats is typically characterized by a more synchronized cohort of preovulatory follicles and ovulation of oocytes that have already reached metaphase II competence. Nevertheless, variability in follicular responsiveness to mating-induced hormonal signals, as well as in the timing and extent of cumulus expansion, has been suggested to influence sperm–oocyte interactions and fertilization success [1]. These features highlight that, although feline oocytes are developmentally more advanced at ovulation than canine oocytes, follicular dynamics remain a critical determinant of reproductive outcome.

Cumulus–oocyte complex (COC) behavior represents another key point of divergence between species. In dogs, cumulus expansion occurs primarily after ovulation and continues during oviductal transit, coinciding with the prolonged period of post-ovulatory oocyte maturation. This delayed expansion may reflect ongoing cytoplasmic reorganization within the oocyte and suggests that somatic cell support extends well beyond ovulation [2,3,27]. In cats, cumulus expansion is closely associated with the preovulatory LH surge and ovulation, consistent with the earlier acquisition of meiotic and cytoplasmic competence [4,6,28]. Despite these differences, the functional role of cumulus cells in regulating cytoplasmic maturation and organelle redistribution in carnivore oocytes remains insufficiently characterized.

From a mechanistic perspective, follicular development in dogs and cats cannot be viewed solely as a process leading to ovulation but must be considered in relation to the acquisition of oocyte developmental competence. Follicle size, timing of luteinization, and the dynamics of cumulus–oocyte interactions likely influence the metabolic and structural maturation of the oocyte. Current assisted reproductive protocols, however, largely extrapolate from other mammalian models [2,3,9] and focus on hormonal control and ovulation timing, with limited consideration of these species-specific follicular features.

Thus, while the general principles of folliculogenesis are conserved, the distinctive ovarian and follicular dynamics of dogs and cats create unique challenges for reproductive management and assisted reproduction. A clearer understanding of how carnivore-specific follicular environments shape oocyte quality is essential for interpreting reproductive failure and for developing more effective, species-appropriate reproductive technologies.

## 3. Oocyte Maturation

### 3.1. Nuclear Maturation

Nuclear maturation refers to the chromosomal and spindle events that effect the transition of the oocyte from the germinal vesicle (GV) stage through germinal vesicle breakdown (GVBD) to metaphase II (MII), the stage competent for fertilization. In many mammals, completion of nuclear maturation in the preovulatory follicle is tightly coupled to acquisition of fertilization competence [7,29]. However, in carnivores the timing of nuclear events differs between species and has important practical consequences for reproductive management.

In the domestic dog, oocytes are ovulated at the GV stage and undergo meiotic resumption during oviductal transit; fertilization is typically possible only 2–3 days after ovulation. This delayed nuclear maturation requires clinicians and ART practitioners to adjust timing of mating or insemination relative to ovulation detection, and it complicates the application of standard in vitro maturation (IVM) protocols that were developed for species in which oocytes are ovulated at more advanced stages [2,8,9].

By contrast, in the domestic cat copulation-induced luteinizing hormone release triggers preovulatory meiotic resumption and ovulation of oocytes largely at the MII stage, allowing a much shorter interval between ovulation and potential fertilization [1,4]. This species difference simplifies timing for some ART procedures in cats but does not eliminate other barriers related to follicular synchrony, cumulus dynamics, and oocyte cytoplasmic readiness.

A critical, and still debated, point is that attainment of an MII spindle or extrusion of the first polar body—conventional markers of nuclear maturation—does not guarantee developmental competence. Increasing evidence indicates that nuclear maturation can be dissociated from the cytoplasmic and metabolic changes required for successful fertilization and early embryogenesis [3,7]. In the context of canine reproduction, this dissociation is especially relevant; because nuclear maturation occurs post-ovulation, standard measurements of meiotic stage may overestimate oocyte quality if not paired with assessments of cytoplasmic maturation.

#### Practical Implications Follow

First, ART timing in dogs must account for delayed nuclear progression and the supporting oviductal environment that facilitates maturation in vivo [2,8]. Second, reliance on nuclear-stage criteria alone for oocyte selection in IVM, IVF, or cryopreservation protocols is likely insufficient; protocols should incorporate or be validated against functional markers of cytoplasmic competence. Finally, comparative differences between dogs and cats caution against direct extrapolation of nuclear stage-based protocols from one species to the other [3,9,30].

### 3.2. Cytoplasmic Maturation

In the context of this review, the term assisted reproductive technologies (ART) encompasses both in vivo approaches, such as ovulation timing and insemination, and in vitro techniques, including in vitro maturation (IVM) and in vitro fertilization (IVF). Importantly, the practical application of these approaches differs markedly between species. In dogs, ART in clinical practice predominantly refers to in vivo reproductive management, whereas IVM and IVF remain largely ineffective. In contrast, in cats, in vitro techniques are more commonly applied and have achieved measurable success. Consequently, cytoplasmic maturation is discussed here as a fundamental biological process with direct relevance to in vitro ART in cats and indirect, but still critical, implications for improving reproductive outcomes in dogs.

Beyond chromosomal segregation and meiotic progression, acquisition of oocyte developmental competence requires extensive cytoplasmic maturation. This process encompasses coordinated changes in organelle number, spatial distribution, and functional state, as well as cytoskeletal remodeling necessary for meiotic division and early embryonic development [7,29]. In dogs and cats, cytoplasmic maturation is of particular importance due to the species-specific dissociation between nuclear maturation and fertilization competence [3,8].

Cytoplasmic maturation involves dynamic reorganization of mitochondria, lipid droplets, endoplasmic reticulum, Golgi apparatus, and cortical granules, processes that are tightly regulated by the cytoskeleton [7,29]. These events ensure adequate energy production, metabolic flexibility, and correct intracellular signaling during fertilization and early cleavage stages. Importantly, cytoplasmic maturation may remain incomplete even when nuclear maturation appears complete, resulting in oocytes that reach the metaphase II stage but lack full developmental potential [3].

In carnivores, available evidence indicates that cytoplasmic maturation follows species-specific temporal and spatial patterns. In dogs, cytoplasmic remodeling continues after ovulation during oviductal transit, coinciding with delayed meiotic progression [2,8]. This prolonged post-ovulatory maturation window suggests that the oviduct provides essential cues for organelle redistribution and metabolic activation that are difficult to replicate under in vitro conditions, contributing to the low efficiency of canine in vitro maturation (IVM) systems [32,33].

In contrast, feline oocytes generally complete both nuclear and cytoplasmic maturation before ovulation following copulation-induced luteinizing hormone release [1,4]. Nevertheless, variability in follicular development, cumulus–oocyte interactions, and ovarian responsiveness can influence cytoplasmic readiness and may contribute to inconsistent reproductive outcomes despite timely nuclear maturation [28,34].

Despite increasing application of in vitro assisted reproductive technologies most protocols continue to rely primarily on nuclear maturation as a surrogate marker of oocyte quality. This approach overlooks the fact that cytoplasmic maturation governs organelle functionality, cytoskeletal integrity, and retention of essential cytoplasmic components within the oocyte during polar body extrusion [7,35]. As a result, oocytes classified as mature based on meiotic criteria alone may still exhibit compromised developmental competence, particularly in species with delayed or prolonged maturation processes.

Ultrastructural and imaging studies in dogs and cats have provided valuable descriptive insights into cytoplasmic organization during oocyte maturation [32,34,36]. However, functional correlations between organelle redistribution, metabolic activation, and fertilization or embryo development remain limited. This gap is especially evident in canine oocytes matured in vitro, which often show discordance between nuclear progression and cytoplasmic organization when compared with oocytes matured in vivo [3,32].

Taken together, cytoplasmic maturation represents a central, yet incompletely understood, determinant of oocyte quality in dogs and cats, with direct implications for in vitro techniques in cats and broader relevance for optimizing reproductive management in dogs. Recognition of its importance provides the conceptual framework for examining specific organelle systems—most notably mitochondria, lipid droplets, and the cytoskeleton—in greater detail in the following section.

## 4. Organelle Specific Mechanisms

### 4.1. Mitochondria

Mitochondria undergo marked changes in number, spatial distribution, and functional state during oocyte growth and maturation across mammalian species [29,37,38,39]. Available evidence indicates that these processes are likewise dynamic in canine and feline oocytes, although species-specific patterns and functional implications remain incompletely understood [32,36].

In both dogs and cats, mitochondrial distribution within the ooplasm has been described as homogeneous, heterogeneous, or peripheral/pericortical, and mitochondria may occur either as dense clusters or in a more dispersed form [32,34,36]. In canine oocytes, immature stages are typically characterized by a homogeneous distribution of dispersed mitochondria. The most pronounced changes occur following germinal vesicle breakdown and progression to the metaphase II stage, when mitochondria are predominantly organized into dense clusters distributed either homogeneously throughout the cytoplasm or peripherally beneath the oolemma [32,33].

Notably, mitochondrial organization differs between in vivo- and in vitro-matured canine oocytes. Oocytes matured in vivo and recovered from the oviduct consistently exhibit homogeneous cytoplasmic distribution of mitochondrial clusters, whereas in vitro-matured oocytes less frequently reach the metaphase II stage. Among those that do, the mitochondrial distribution is often heterogeneous or peripherally clustered, suggesting incomplete cytoplasmic maturation despite nuclear progression [32]. These observations support the view that mitochondrial redistribution accompanies meiotic maturation but may not be fully achieved under in vitro conditions.

In feline oocytes, immature stages predominantly display peripheral, non-uniform mitochondrial distribution, frequently forming dense clusters [28,36]. During in vitro maturation, mitochondria remain largely cortical at the GVBD stage but appear less clustered [28,34,36,40]. In contrast, in vivo-matured feline oocytes show predominantly homogeneous mitochondrial distribution, with mixed patterns observed less frequently [34]. While these spatial changes parallel meiotic progression, direct evidence linking mitochondrial redistribution to functional activation remains limited.

Collectively, available data indicate that mitochondrial redistribution toward a more homogeneous cytoplasmic organization correlates with meiotic maturation in both species and is more efficiently achieved in vivo than in vitro. In dogs, delayed or incomplete mitochondrial maturation appears to represent a major cellular constraint on oocyte competence, whereas in cats it constitutes an important, though not exclusive, contributing factor alongside other metabolic and environmental influences.

### 4.2. Mitochondrial DNA Copy Number

In several mammalian species, mitochondrial DNA (mtDNA) copy number has been proposed as a proxy for mitochondrial biogenesis during oogenesis and has been linked to developmental competence [38,41]. This parameter remains poorly characterized in carnivores. Studies in dogs have largely focused on ultrastructural and spatial aspects of mitochondria without direct quantification of mtDNA content, while feline data are limited and often combine oocyte and embryo stages.

In cats, mtDNA copy number has been reported to increase sharply from approximately 1–5 × 10^4^ in pre-antral and early antral stages to 2–3 × 10^5^ in late antral oocytes [28]. This pattern resembles that observed in other mammals and is consistent with the “mitochondrial bottleneck” theory, which proposes selective amplification of mtDNA to maintain genomic integrity [41,42]. To date, comparable quantitative data are lacking for canine oocytes, and the timing and regulation of mtDNA amplification in this species remain unknown.

Although higher mtDNA copy number has been associated with improved fertilization outcomes in human oocytes [41] and declines with maternal age [43], it is unclear whether similar relationships exist in dogs and cats. As a result, the functional significance of mtDNA copy number in determining oocyte competence in carnivores remains largely speculative.

Ultrastructural studies in feline [44,45] and canine oocytes [27] further indicate close physical associations between mitochondria and lipid droplets, with some mitochondria directly attached to lipid droplet surfaces. It has been hypothesized that these mitochondria may function analogously to peridroplet mitochondria described in other tissues, participating in lipid synthesis and expansion rather than lipid utilization [46,47,48]. Whether such mechanisms operate in carnivore oocytes and how they relate to mitochondrial redistribution during maturation require further investigation.

### 4.3. Lipid Droplets

Lipid droplets (LDs) are increasingly recognized as dynamic organelles involved in lipid storage, synthesis, and metabolic regulation rather than passive energy reservoirs [49,50]. In oocytes, LDs interact extensively with the endoplasmic reticulum, Golgi apparatus, and mitochondria, forming an integrated metabolic network that supports cytoplasmic maturation.

Canine oocytes are distinguished by their lipid-rich cytoplasm, first described ultrastructurally by Guraya [51] and later by Tesoriero (1982) [52]. The accumulation of large numbers of LDs during follicular growth represents one of the earliest morphological indicators of oocyte maturation in this species [51,52]. Despite these observations, direct analyses of lipid composition in canine oocytes remain scarce. A comparative MALDI-MS study identified phosphatidylcholine as the most abundant lipid class in canine, bovine, and feline oocytes, with interspecies differences primarily reflecting relative proportions rather than distinct lipid classes [53].

Stage-dependent changes in LD distribution have been reported in canine oocytes collected during different phases of the estrous cycle. During anestrus, LDs predominantly localize to the perinuclear region, whereas during the follicular phase they exhibit a more diffuse cytoplasmic distribution. In the luteal phase, LDs are mainly positioned peripherally within the cortical region [54]. Whether these spatial changes are accompanied by alterations in lipid composition or metabolic function remains unknown.

In feline oocytes, lipid metabolism and LD dynamics have been examined more extensively. Comparative analyses of immature and in vitro-matured oocytes revealed differences in sterol esterification and monoacylglycerol content, as well as stage-dependent variation in expression of genes associated with lipid metabolism [55]. However, results regarding total LD abundance have been inconsistent. While one study reported ambiguous changes in LD quantity following in vitro maturation [55], another using confocal microscopy demonstrated a significant increase in LD number and total volume, accompanied by reduced LD diameter and altered expression of lipid metabolism-related genes [40].

LD distribution also changes during feline oocyte maturation. Pre-IVM oocytes show no dominant pattern, whereas GV-stage IVM oocytes exhibit predominantly diffuse LD distribution, and MII-stage oocytes display almost exclusively diffuse patterns [40]. These findings suggest active LD remodeling during cytoplasmic maturation, potentially reflecting shifts in lipid synthesis, storage, and utilization.

From an applied perspective, LD abundance has important implications for assisted reproduction. High LD content has been associated with increased susceptibility to cryodamage during vitrification [56,57]. Conversely, experimental modulation of lipid metabolism using supplements such as L-carnitine, forskolin, or conjugated linoleic acid has shown promise in reducing LD content during in vitro maturation, although effects on developmental competence remain unclear [55]. Notably, reduced LD content in pre-IVM oocytes has also been linked to lower maturation rates, suggesting that both excessive and insufficient lipid reserves may be detrimental [58].

Overall, LDs undergo species- and stage-specific remodeling during oocyte maturation in dogs and cats. However, the lack of integrated ultrastructural, compositional, and functional studies—particularly in dogs—currently limits the rational optimization of IVM and other assisted reproductive techniques.

### 4.4. Cytoskeleton

The cytoskeleton is a key integrative system coordinating nuclear maturation, cytoplasmic organization, and asymmetric division during oocyte meiosis. Actin filaments and microtubules regulate spindle assembly and positioning, polar body extrusion, and spatial retention of cytoplasmic components essential for early embryogenesis [54,59].

Actin-mediated spindle migration toward the oocyte cortex enables the highly asymmetric cytokinesis characteristic of polar body extrusion, while microtubules ensure accurate chromosome alignment and segregation [54,59]. Disruption of these processes can result in spindle mispositioning, abnormal polar body formation, and loss of cytoplasmic material, ultimately compromising oocyte developmental competence.

Although most mechanistic insights into oocyte cytoskeletal dynamics derive from rodent and livestock models, available evidence suggests that similar principles apply in carnivores, with important species-specific implications. In dogs, meiotic resumption and polar body extrusion occur post-ovulation during oviductal transit, implying that cytoskeletal remodeling extends beyond the follicular environment. This prolonged maturation period may increase reliance on stable actin and microtubule dynamics under in vivo conditions that are difficult to replicate during in vitro maturation.

Cytoskeletal elements also contribute to organelle positioning and redistribution within the ooplasm. Actin networks are involved in anchoring mitochondria and lipid droplets, facilitating the establishment of a homogeneous cytoplasmic environment prior to fertilization [7,29]. Incomplete cytoskeletal remodeling may therefore indirectly impair mitochondrial clustering, lipid metabolism, and overall cytoplasmic competence, even when nuclear maturation appears complete.

In cats, preovulatory meiotic resumption suggests that cytoskeletal reorganization largely occurs within the follicle. Nevertheless, variability in follicular synchrony and cumulus–oocyte interactions may influence cytoskeletal regulation and organelle retention, potentially affecting oocyte quality. Direct experimental evidence addressing these mechanisms in feline oocytes remains limited.

From an assisted reproduction perspective, cytoskeletal integrity represents a critical but underappreciated determinant of oocyte quality. Standard IVM protocols primarily target hormonal and meiotic endpoints, with limited attention to actin and microtubule dynamics. Mechanical stress, temperature fluctuations, or suboptimal culture conditions may destabilize the cytoskeleton, increasing the risk of spindle abnormalities and cytoplasmic loss, particularly in lipid-rich canine oocytes and during procedures such as vitrification.

Taken together, cytoskeletal dynamics link nuclear maturation, organelle redistribution, and asymmetric division into a unified process underlying oocyte developmental competence. In dogs and cats, species-specific timing of meiotic and cytoplasmic events suggests that improved understanding of cytoskeletal regulation is essential for advancing both basic reproductive biology and the optimization of assisted reproductive technologies.

### 4.5. Molecular Pathways in Cytoskeleton Remodeling

Generally, the molecular pathways involved in cytoskeleton remodeling during oocyte maturation have been described primarily on the basis of research conducted in mammalian species other than cats and dogs. To the best of our knowledge, the pathways outlined below have not been experimentally validated, even partially, in canine or feline oocytes. Nevertheless, the core molecular components involved—particularly small GTPases and actin nucleators—have been functionally characterized across multiple mammalian models with broadly conserved effects. It is therefore plausible that similar regulatory mechanisms operate in carnivore oocytes, although this assumption requires direct experimental verification.

**Figure 3:**
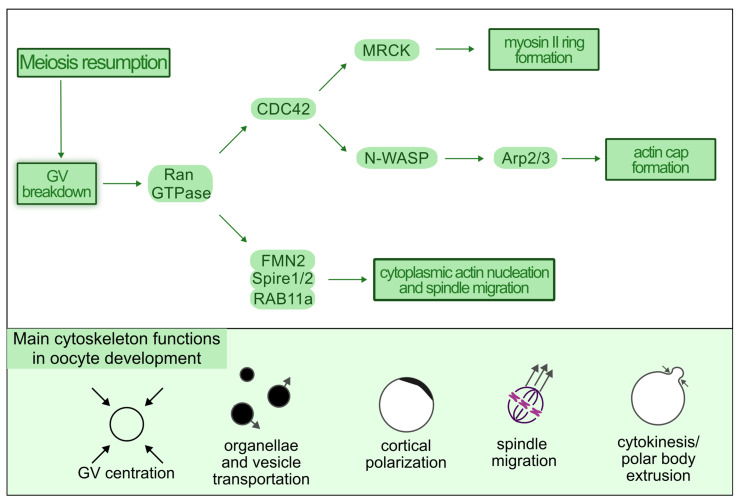
A summary of the major roles of the cytoskeleton during oocyte meiosis and the pathways it affects. Meiosis resumption leads to nucleolemma breakdown (GVBD), releasing Ran GTPase. This activates two pathways—via CDC42 and nucleators (FMN2 and Spire 1/2). The CDC42-dependent pathway is necessary for the formation of the actin cap and surrounding myosin ring. Once activated, FMN2 and Spire1/2 are recruited to Rab11a + vesicles, which then function as nucleation vesicles. This allows creation of new actin filaments, which is necessary for meiotic spindle migration.

Cytoskeletal remodeling during oocyte maturation is orchestrated by a network of conserved molecular pathways involving members of the small GTPase superfamily, including Rho-, Ran-, and Rab-family proteins [60]. These regulators coordinate actin and microtubule dynamics required for spindle positioning, asymmetric cytokinesis, and polar body extrusion Figure 3).

Among these, Ran GTPase plays a central role [61]. Under interphase conditions, Ran regulates nucleocytoplasmic transport by mediating the release of nuclear localization signal (NLS)-containing cargo from importins within the nucleus and facilitating importin recycling to the cytoplasm [62]. Following meiotic resumption and germinal vesicle breakdown (GVBD), Ran adopts a distinct spatial function. Chromatin-associated Ran-GTP establishes a concentration gradient centered around the meiotic chromosomes, which serves as a positional cue for cytoskeletal organization [53].

This Ran-GTP gradient promotes the cortical recruitment of CDC42, a Rho-family GTPase that initiates localized actin remodeling. Activation of CDC42 triggers the CDC42–N-WASP–ARP2/3 pathway, which drives branched actin polymerization and is essential for formation of the cortical actin cap that determines the site of polar body extrusion [63]. In parallel, CDC42 activates MRCK (myotonic dystrophy kinase-related Cdc42-binding kinase), leading to activation of non-muscle myosin II and formation of a contractile actomyosin ring surrounding the actin cap [64,65]. Together, these processes ensure asymmetric division while preserving the bulk of cytoplasmic content within the oocyte.

Beyond cortical remodeling, Ran-GTP also regulates cytoplasmic actin dynamics required for meiotic spindle migration and positioning. Elevated Ran activity near the spindle inhibits importin-mediated sequestration of Formin-2 (FMN2), allowing FMN2 to interact with Spire1/2. These actin nucleators associate with Rab11a-positive vesicles, which function as cytoplasmic actin nucleation platforms [66,67]. As a result, actin filaments preferentially nucleate in the vicinity of the spindle, generating forces that promote its migration toward the oocyte cortex.

Although the involvement of FMN2 and Spire1/2 in spindle migration is well established—disruption of either results in defective spindle positioning—the precise mechanical mechanisms underlying this process remain incompletely resolved [68]. Current models propose two non-mutually exclusive mechanisms: (i) generation of cytoplasmic flow driven by ARP2/3-mediated cortical actin thickening that passively displaces the spindle toward the oolemma, and (ii) active myosin-dependent pulling forces that draw one spindle pole toward the cortex [69].

Recent evidence further suggests that actin regulatory components may influence meiotic competence under assisted reproductive conditions. For example, the presence of the ARP2/3 complex in feline follicular fluid-derived extracellular vesicles has been proposed to contribute to higher rates of meiosis resumption in vitrified feline oocytes, highlighting a potential link between extracellular signaling, actin regulation, and oocyte maturation efficiency [70]. While these findings are preliminary and species-specific, they support the concept that modulation of actin-regulatory pathways may influence oocyte competence under in vitro conditions.

Importantly, most mechanistic insights into Ran-, CDC42-, and actin nucleator-dependent pathways derive from murine and other non-carnivore models. Direct functional validation of these pathways in canine and feline oocytes remains limited. Given the prolonged and species-specific timing of meiotic and cytoplasmic maturation in carnivores, altered kinetics or regulation of cytoskeletal remodeling may contribute to the marked sensitivity of canine oocytes to in vitro manipulation and to the frequent discordance between nuclear and cytoplasmic maturation. Targeted investigation of these molecular regulators in dog and cat oocytes is therefore essential to determine their relevance for improving oocyte quality and optimizing assisted reproductive strategies in these species.

## 5. Implications in ART

Despite increasing clinical use of assisted reproductive technologies (ART) in selected areas of small animal practice, success rates in dogs and, to a lesser extent, cats remain variable and often suboptimal. The species-specific patterns of oocyte maturation and cytoplasmic remodeling described in the preceding sections highlight several biological constraints that limit the effectiveness of protocols largely extrapolated from other mammalian models.

### 5.1. Timing of ART and Oocyte Selection

In dogs, ovulation of oocytes at the germinal vesicle stage and the requirement for prolonged post-ovulatory maturation pose fundamental challenges for accurate timing of mating, artificial insemination, and oocyte collection for in vitro procedures [2,8,9]. Reliance on ovulation timing or nuclear stage alone may result in selection of oocytes that have progressed meiotically but have not yet completed cytoplasmic maturation. In contrast, feline oocytes are generally ovulated at metaphase II following copulation-induced luteinizing hormone release, allowing a narrower fertilization window but greater predictability with respect to nuclear maturity [1,4]. These differences underscore the need for species-specific criteria for oocyte selection and ART scheduling.

### 5.2. IVM and Cytoplasmic Competence

In vitro maturation (IVM) remains a major bottleneck in canine reproduction. Comparative studies demonstrate that canine oocytes matured in vivo exhibit more complete mitochondrial redistribution and cytoplasmic organization than those matured in vitro, even when nuclear maturation appears comparable [32,33]. Similar, though less pronounced, discrepancies have been observed in feline oocytes [34]. These findings indicate that current IVM systems fail to replicate key aspects of the follicular and oviductal environments required for coordinated cytoplasmic maturation. Optimization of in vitro maturation (IVM) protocols will therefore require greater attention to metabolic support, organelle dynamics, and cytoskeletal stability rather than focusing solely on hormonal or meiotic endpoints.

### 5.3. Mitochondrial Function and Metabolic Support

Mitochondrial redistribution and biogenesis are closely linked to oocyte developmental competence in both species. In dogs, delayed or incomplete mitochondrial maturation appears to represent a central cellular constraint, particularly under in vitro conditions [3,32]. Although mtDNA copy number has been associated with fertilization potential in human oocytes [41], comparable functional data are lacking in carnivores, limiting its immediate application as a biomarker. Nevertheless, these observations suggest that ART protocols should aim to support mitochondrial function and metabolic readiness, potentially through culture conditions that better mimic in vivo maturation environments.

### 5.4. Lipid Droplets and Cryopreservation Strategies

The high lipid content of canine oocytes and the dynamic remodeling of lipid droplets during maturation have important implications for cryopreservation. Elevated lipid abundance has been associated with increased susceptibility to cryodamage during vitrification [56,57]. Conversely, insufficient lipid reserves have been linked to reduced maturation rates, indicating that both excess and deficiency may compromise oocyte quality [58]. Experimental approaches aimed at modulating lipid metabolism during IVM, including supplementation with L-carnitine, forskolin, or conjugated linoleic acid, have shown variable effects on lipid content and maturation outcomes [55]. These findings highlight the need for balanced, species-specific strategies when targeting lipid metabolism in ART.

### 5.5. Cytoskeletal Integrity and Technical Manipulation

Cytoskeletal stability is essential for successful meiotic progression, organelle retention, and asymmetric division. Mechanical stress, temperature fluctuations, and suboptimal culture conditions may disrupt actin and microtubule dynamics, leading to spindle abnormalities and cytoplasmic loss [54,71]. Such effects may be particularly pronounced in canine oocytes, where meiotic and cytoplasmic maturation occur over an extended post-ovulatory period. Greater consideration of cytoskeletal integrity during oocyte handling, maturation, and cryopreservation may therefore improve ART outcomes.

### 5.6. Species-Specific ART Optimization

Collectively, available evidence indicates that the limited success of ART in cats and the constrained effectiveness of advanced reproductive technologies in dogs reflects not a single technical limitation but the cumulative impact of species-specific oocyte biology. Approaches that prioritize nuclear maturation alone are unlikely to yield substantial improvements in fertilization or embryo development. Instead, future optimization of ART protocols should integrate assessments of cytoplasmic maturation, organelle organization, metabolic readiness, and cytoskeletal stability. Advancing this integrative perspective will require coordinated basic and applied research aimed at defining functional markers of oocyte competence tailored specifically to carnivore reproduction.

## 6. Conclusions

Reproductive inefficiency in dogs and cats remains a persistent challenge despite substantial advances in veterinary reproductive medicine and assisted reproductive technologies. While endocrine regulation, timing of ovulation, sperm quality, uterine receptivity, and early embryonic survival all contribute to the reproductive outcome, growing evidence indicates that oocyte developmental competence represents a critical biological constraint that has not yet been fully integrated into clinical and experimental practice.

This review highlights that oocyte competence cannot be adequately assessed based on nuclear maturation alone. In carnivores, and particularly in the dog, the dissociation between meiotic progression and cytoplasmic maturation complicates the interpretation of conventional maturity markers and limits the predictive value of nuclear stage for fertilization and embryo development. Ultrastructural studies in dogs and cats consistently demonstrate that organelle redistribution, metabolic preparedness, and cytoskeletal organization undergo species-specific and temporally distinct changes that are not reliably reproduced under current in vitro conditions.

At the same time, the available literature remains largely descriptive, and definitive functional links between specific ultrastructural features and reproductive success are still lacking. Mitochondrial distribution patterns, lipid droplet remodeling, and cytoskeletal dynamics have been associated with meiotic progression and oocyte quality, yet their causal relationships with fertilization competence and embryonic development remain incompletely defined. This limitation underscores the need for caution when interpreting ultrastructural observations as direct indicators of developmental potential. Future research should therefore prioritize functional validation studies that correlate specific ultrastructural parameters with fertilization rates, embryo development, and live birth outcomes in well-defined experimental settings.

Importantly, ultrastructural immaturity should not be viewed as an isolated or overriding cause of reproductive failure in dogs and cats. Rather, it represents one component of a multifactorial system in which endocrine signaling, follicular environment, gamete interactions, and uterine factors collectively shape reproductive outcomes. The relative contribution of oocyte ultrastructure likely varies between species, reproductive contexts, and assisted reproductive techniques, and should therefore be evaluated within an integrated biological framework. Standardized criteria for assessing cytoplasmic maturation and organelle organization across laboratories would further enhance comparability and reproducibility of findings.

From a translational perspective, the findings summarized in this review suggest that further progress in carnivore assisted reproduction will require a shift away from protocols based primarily on hormonal control and nuclear-stage assessment. Greater emphasis should be placed on understanding and supporting cytoplasmic maturation, organelle functionality, and cytoskeletal stability, particularly in the context of in vitro maturation, cryopreservation, and other manipulative procedures. However, meaningful optimization of these approaches will depend on future studies that move beyond descriptive ultrastructural analysis toward functional and outcome-based validation. Integration of advanced imaging, metabolic profiling, and molecular assays with clinically relevant endpoints may help bridge the gap between cellular observations and practical ART success.

In conclusion, the oocyte ultrastructure provides a valuable lens through which to examine the limitations of current reproductive technologies in dogs and cats, but it should be considered as part of a broader, integrative view of reproductive biology. Advancing the field will require coordinated basic and applied research aimed at defining species-specific determinants of oocyte competence and translating this knowledge into biologically informed, clinically effective reproductive strategies. Such efforts will be essential to move from descriptive characterization toward predictive and intervention-oriented models of oocyte quality in companion animals.

## Data Availability

No new data were created or analyzed in this study.

## Abbreviations

AMP	non-cyclic AMP
AP-3	AP-3 adaptor complex
ARP2/3	ARP2/3 complex
ART	Assisted Reproductive Technologies
BMP15	bone morphogenetic protein 15
cAMP	cyclic adenosine monophosphate
CCs	cumulus cells
CDC42	Cell Cycle Division Rho GTPase
cGMP	cyclic GMP
CL	corpus luteum
COC	cumulus–oocyte complex
CX37	connexin 37
CX43	connexin 43
EGF	EGF receptors
ER	endoplasmic reticulum
EVs	extracellular vesicles
ffEVs	follicular fluid extracellular vesicles
FMN2	formin-2
GDF9	growth differentiation factor 9
GFAP	glial fibrillary acidic protein
GV	germinal vesicle
GVBD	germinal vesicle breakdown
IFs	Intermediate filaments
IVM	in vitro maturation
LH	luteinizing hormone
MGCs	mural granulosa cells
MI	metaphase I
MII	metaphase II
MPF	maturation-promoting factor
MRCK	Myotonic Dystrophy Kinase-Related Cdc42-Binding Protein
NF-L	type of intermediate filament
NLS	Nuclear Localization Signal
NPC1	Niemann-Pick type C1
NPPC	peptide produced by MGCs
NPR2	NPR2 receptor
N-WASP	Neural wiskott-Aldrich Syndrome Protein
OSFs	oocyte-secreted factors
PDE3	phosphodiesterase PDE3
PDE3A	phosphodiesterase 3A
PDM	peri-droplet mitochondria
PKA	kinase PKA
PVS	perivitelline space
Rab	Rab GTPases
SCNT	somatic cell nuclear transfer
SNARE	fusion component
TZPs	transzonal projections
ZP	zona pellucida
